# Supplementary material for: Isolation and Characterization of Poeciguamerin, a Peptide with Dual Analgesic and Anti-Thrombotic Activity from the Poecilobdella manillensis Leech
Source: Int J Mol Sci. 2023 Jul 4;24(13):11097. doi: 10.3390/ijms241311097 (PMC10342134; doi:10.3390/ijms241311097)
Supplement: Supplementary file 1 [file ijms-24-11097-s001.zip › ijms-2452020-supplementary.pdf]

Supplementary Information

1. Supplementary Methods

1.1. Expression, purification, and activity identification of poeciguamerin

The nucleotide sequence was predicted to express the complete poeciguamerin, and the nucleotide sequence was optimized according to the *E. coli* expression preference codon. The pET-32a (+) vector (Novagen) was selected, and the plasmid was constructed with a TEV protease cleavage site (amino acid sequence: ENLYFQG) inserted between the His tag and target sequence to facilitate cleavage and purification after fusion expression. The constructed plasmids were transformed into E. coli BL21 (DE3) host cells. The transformed cells were incubated at 37 °C for 1 h, then plated onto LB solid culture plates containing 100 μg/ml ampicillin (Amresco, Biosharp) and incubated at 37 °C for 16 h. The resulting colonies were selected for sequencing to identify successful constructs. Colonies were picked for sequencing and the correct colonies were considered successful. Correctly sequenced strains were inoculated into liquid LB medium containing 100 μg/ml ampicillin and incubated for 10 h at 37 °C with shaking at 200 rpm. The cultured bacterial broth was inoculated into fresh Luria-Bertani (LB) medium at a ratio of 1:500 for expansion and incubated for 8 h at 37 °C with shaking at 200 rpm. At an optical density (OD_{600}) of approximately 0.6, isopropyl-β-D-thiogalactoside (IPTG, Macklin) was added at a final concentration of 1 mM and induced for 6 h at 28 °C with shaking at 80 rpm. Bacteria were harvested by centrifugation at 8 000 rpm for 3 min. Bacteria were resuspended in binding buffer (20 mM Tris, 100 mM NaCl, pH 8.0) and cells were disrupted by sonication (3 s sonication, 3 s hold, 20 min total). The supernatant was collected by

centrifugation (12 000 ×*g* for 2 h at 4 ℃) and filtered through a 0.45-μm membrane. The His-tagged fusion protein was purified using Ni-TED Sefinose (C610030-0100, BBI). The fusion protein solution was replaced with enzyme digestion buffer (50 mM NaH_2PO_4, 150 mM NaCl, pH 8.0) using 3K ultrafiltration tubes, with a rTEV (E8350, Solarbio) concentration of 5 U/μl. The reagents were mixed according to the instructions: 4 μl of 250 × rTEV protease buffer and 2 μl of rTEV protease were added per 1 000 μg of fusion protein and mixed well, with the volume fixed to 1 000 μl using enzymatic digestion buffer. The mixture was digested at 28 ℃ for 14 h. The digested proteins were separated, purified, and identified according to the methods described in Sections 4.2 and 4.3.

2. Supplementary Figures

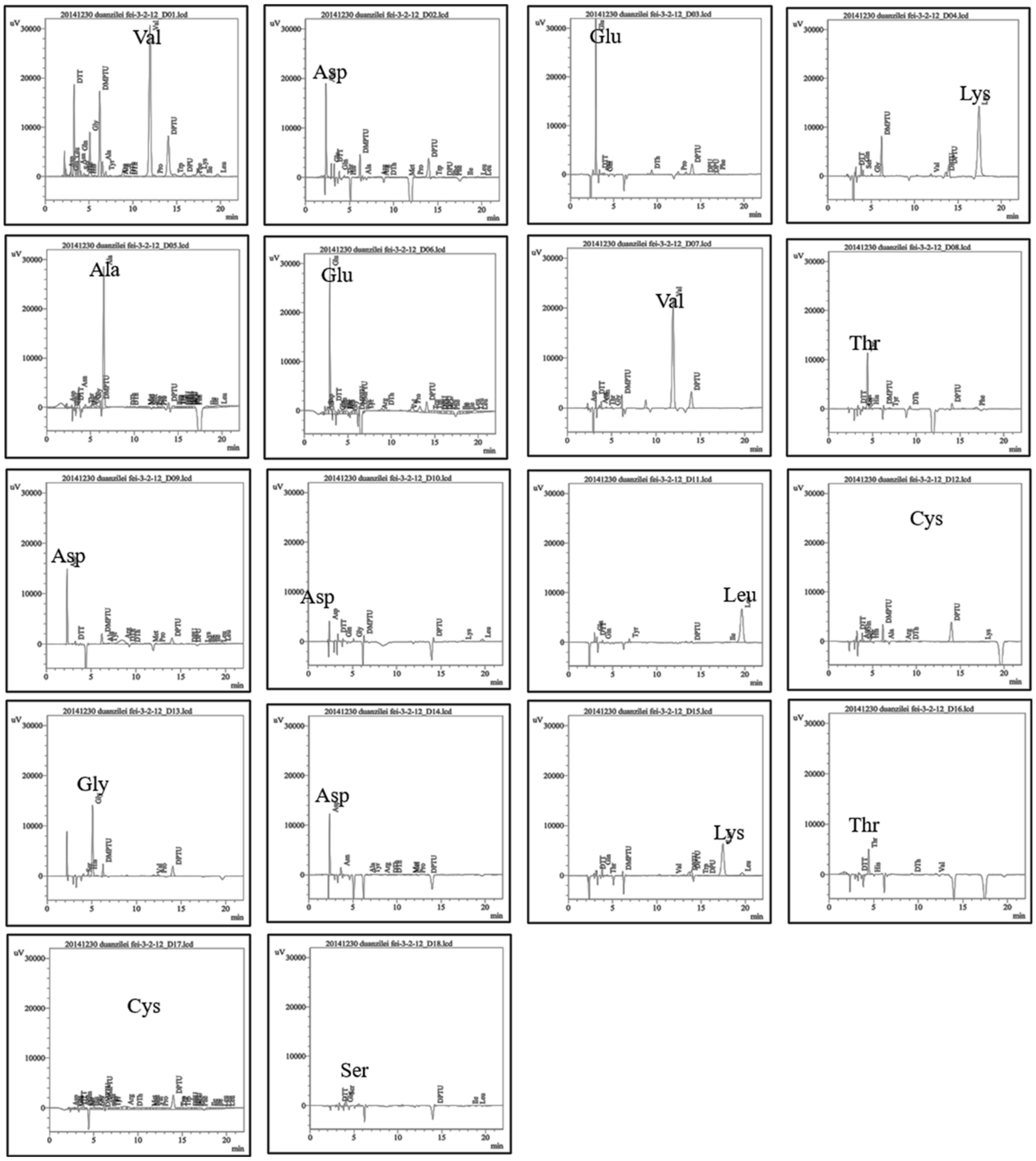

Figure S2. The Edelman degradation method for peptide sequence detection.

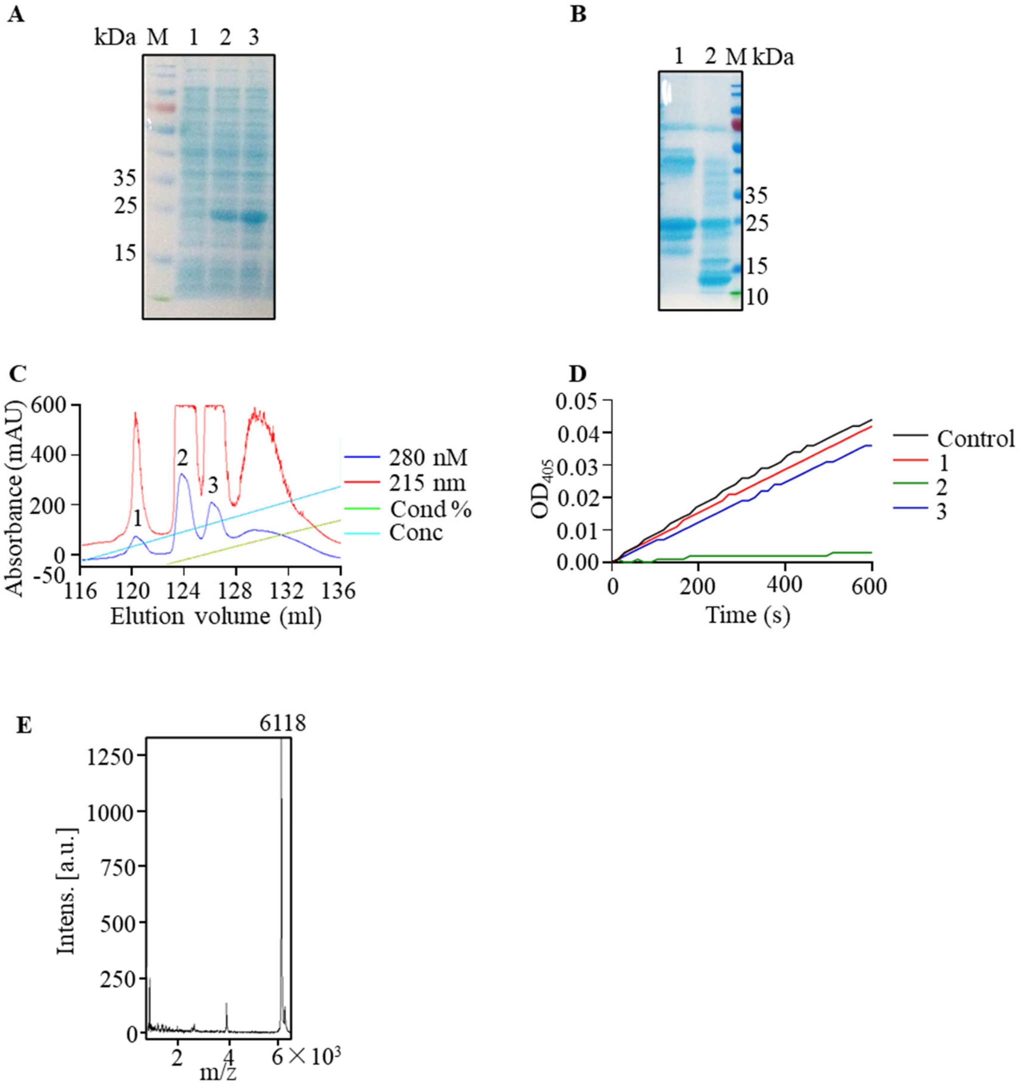

Figure S2. Prokaryotic expression and purification of poeciguamerin. **(A)** Expression levels of poeciguamerin before and after induction of prokaryotic expression by IPTG were analyzed by Coomassie Brilliant Blue staining. **(B)** Analysis of the effects of enterokinase cleavage of SPINKL6-his by Coomassie Brilliant Blue staining. **(C)** Efficiency of purification of poeciguamerin by HPLC separation. **(D)** Inhibition of elastase bioactivity by enzyme kinetic analysis of each protein peak in HPLC. **(E)** Molecular weight of second protein peak determined by MALDI-TOF MS.
